# Supplementary material for: Association of Blood Leukocytes and Hemoglobin with Hospital Mortality in Acute Pulmonary Embolism
Source: J Clin Med. 2023 Sep 28;12(19):6269. doi: 10.3390/jcm12196269 (PMC10573828; doi:10.3390/jcm12196269)
Supplement: Supplementary file 1 [file jcm-12-06269-s001.zip › jcm-2601822-supplementary.pdf]

Supplementary Materials

Association of blood leukocytes and hemoglobin with hospital mortality in acute pulmonary embolism

Obradovic Slobodan[1,2], Dzudovic Boris[2,3]*, Subotic Bojana[1], Salinger Sonja[4,5], Matijasevic Jovan[6,7], Benic Marija[6], Kovacevic Tamara[8,9], Kovacevic-Kuzmanovic Ana[10], Mitevska Irena[11], Miloradovic Vladimir[13,14], Jevtic Ema[13], and Neskovic Aleksandar[15,16]

1 Clinic of Cardiology, Military Medical Academy of Belgrade, Serbia
2 School of Medicine, University of Defense, Belgrade, Serbia
3 Clinic of Emergency Internal Medicine, Military Medical Academy, Belgrade, Serbia
4 Clinic of Cardiology, Clinical Center Nis, Serbia
5 School of Medicine, University of Nis, Serbia
6 Institute of Pulmonary Diseases Vojvodina, Novi Sad, Serbia
7 School of Medicine, University of Novi Sad, Serbia
8 Clinic of Cardiology, Clinical Center Banja Luka, Bosnia and Herzegovina
9 School of Medicine, University of Banja Luka, Bosnia and Herzegovina
10 General Hospital Pancevo, Serbia
11 University Cardiology Clinic, Intensive Care Unit, Skopje, North Macedonia
12 Clinic of Cardiology, Clinical Center Kragujevac, Serbia,
13 School of Medicine, University of Kragujevac, Serbia
14 Clinic of Cardiology, University Clinical Center Zemun, Serbia
15 School of Medicine, University of Belgrade, Serbia
* Correspondence: dzuda1977@gmail.com (BDZ)

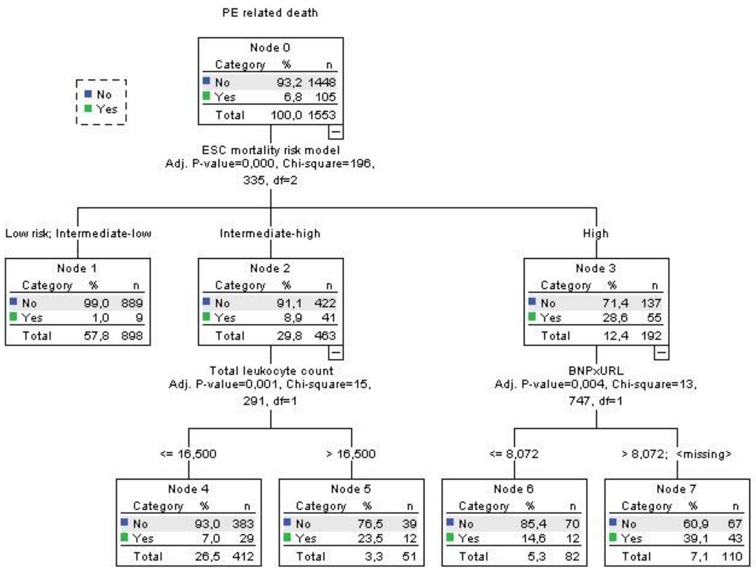

Figure S1. Decision tree using ESC mortality risk model, TLC, and BNP or NTproBNP x URL.

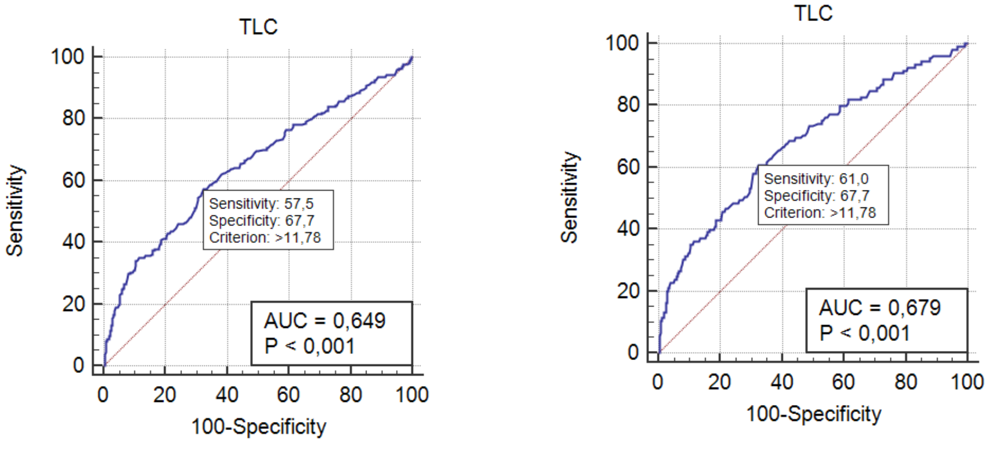

(**a**) (**b**)

Figure S2. ROC curves for TLC for the prediction of all-cause (panel a) and PE related (panel b) hospital death. TLC level of >11.78x109/L has PPV=17,6% and NPV = 93% for all-cause death and PPV=12,1% and NPV = 96% for PE related hospital death.

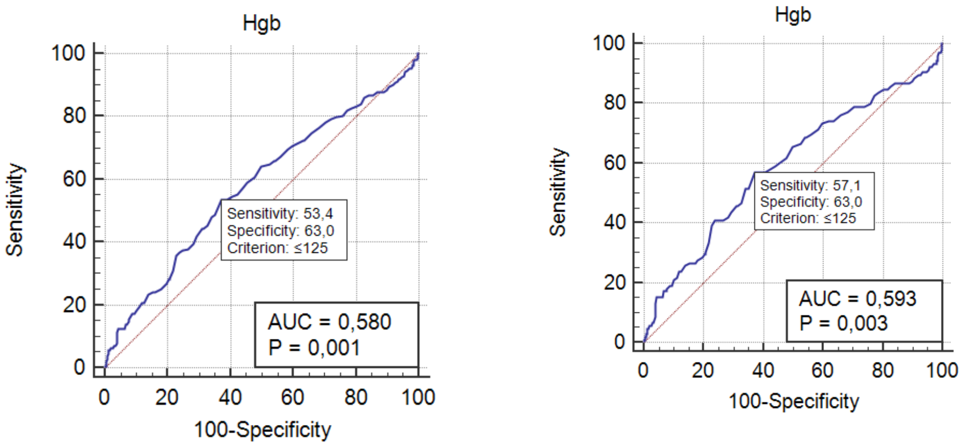

(**a**) (**b**)

Figure S3. ROC curves for Hb levels for the prediction of all-cause (panel a) and PE related (panel b) hospital death. TLC level of >11.78x109/L has PPV=17,6% and NPV = 93% for all-cause death and PPV=12,1% and NPV = 96% for PE related hospital death.
